# The exposure rate to hepatitis B and C viruses among medical waste handlers in three government hospitals, southern Ethiopia

**DOI:** 10.4178/epih/e2016001

**Published:** 2016-01-05

**Authors:** Anteneh Amsalu, Mesfin Worku, Endale Tadesse, Techalew Shimelis

**Affiliations:** 1 Department of Medical Microbiology, School of Biomedical and Laboratory Sciences, University of Gondar, Gondar, Ethiopia; 2 Department of Medical Laboratory Science, College of Medicine and Health Sciences, Hawassa University, Hawassa, Ethiopia

**Keywords:** Hepatitis B virus, Hepatitis C virus, Medical waste handlers, Vaccination

## Abstract

**OBJECTIVES:**

The aim of this study was to assess the rate of and risk factors for exposure to hepatitis B virus (HBV) and hepatitis C virus (HCV) among medical waste handlers.

**METHODS:**

A cross-sectional study was conducted from December 2014 to January 2015. A total of 152 medical waste handlers (MWH) and 82 non-medical waste handlers (NMWH) were studied. Serum samples were collected from participants and screened for hepatitis B surface antigen (HBsAg), hepatitis B core antibody (anti-HBc) and anti-HCV using rapid immunochromatography assay. MWH were also screened for hepatitis B surface antibody (anti-HBs).

**RESULTS:**

The respective prevalence of HBsAg, anti-HBc and anti-HCV was 1.3%, 39.4%, and 0.7% in MWH, compared to 2.4%, 17.1%, and 1.2%, respectively, in NMWH. Among MWH, 58.6% were susceptible to HBV infection. There was a significant difference in the rate of lifetime exposure to HBV in MWH compared with NMWH (odds ratio [OR], 3.17; 95% confidence interval [CI], 1.64 to 6.13). However, there was no significant difference between participant groups with respect to current HBV infection (OR, 0.53; 95%CI, 0.07 to 3.86) or anti-HCV (OR, 0.54; 95%CI, 0.03 to 8.69). Age older than 40 years and working in a hospital laundry were independent predictors of lifetime exposure to HBV infection. Eleven (7.2%) respondents were vaccinated against HBV.

**CONCLUSIONS:**

Lifetime exposure to HBV infection was significantly higher in MWH than in NMWH. The majority of MWH was not vaccinated against HBV and thus remains susceptible to contracting the infection. Screening upon hire followed by vaccination of MWH is recommended to reduce the transmission of HBV.

## INTRODUCTION

Waste produced in the course of healthcare activity carries a high potential for infection, and its poor management poses direct and indirect health risks to health care workers (e.g., doctors, nurses, and laboratory workers), waste handlers, and patients in hospitals [1]. Medical waste contains wide range of pathogens such as human immunodeficiency virus (HIV), hepatitis B virus (HBV), and hepatitis C virus (HCV) [2]. According to the World Health Organization, around 500 million people worldwide are chronically infected with either HBV or HCV. An estimated 1 million people die each year from causes related to viral hepatitis, most commonly liver disease, including liver cancer. About 57% of cases of liver cirrhosis and 78% of cases of primary liver cancer result from HBV and HCV infection [3]. A varying prevalence of HBV has been documented in different regions of the world; China, South-East Asia, sub-Saharan Africa, most Pacific Islands, and the Amazon basin are most affected. The prevalence of HBV infection in Africa is on average more than 10%, classifying the region as one of high endemicity for HBV [4,5].

HBV is more infectious than other blood-borne viral pathogens and is about 100 times more infectious than HIV. The infectiousness of HBV is partly explained by its higher viral load in the blood, longer viability in the environment (>7 days at room temperature), and transmissibility in the absence of visible blood [6]. Consequently, HBV presents the greatest occupational risk to non-immune health care workers (HCW) [7]. The seroprevalence of hepatitis B surface antigen (HBsAg) among HCW was reported to be 8.1% in Uganda [8] and 9.7% in Ethiopia [9]. It was also reported that medical waste handlers (MWH) are more exposed to HBV infection than other HCW [10], non-medical waste handlers (NMWH) [11,12], or the general population [6]. Indirect inoculation through improperly collected and/or segregated sharp materials is considered to be an occupational hazard for MWH in health institutions, compared to NMWH or the proportion of the general population that has no direct or indirect contact with medical waste [6,12].

Despite the availability of antiviral agents to treat chronic hepatitis, treatment policies are lacking in Ethiopia, and these drugs are inaccessible and very expensive. Therefore, prevention remains of crucial importance. In this regard, appropriate utilization of personal protective equipment (PPE), screening at the time of employment, and vaccinating susceptible individuals at increased risk of infection are direly needed. However, the government still lacks a policy for pre-employment vaccination and post-exposure prophylaxis against HBV for HCW at higher risk of exposure to blood and body fluids [12]. Evidence is scarce concerning the rate of exposure to HBV and HCV infection and potential risk factors among MWH in Ethiopia. Therefore, this study aimed to assess the rate of exposure to HBV and HCV infection, and associated risk factors, in MWH at three government hospitals in southern Ethiopia. Evidence from this study may be presented to public health officials, decision-makers, and other concerned bodies in order to plan possible intervention measures.

## MATERIALS AND METHODS

### Study design and location

A cross-sectional study was conducted from December 2014 to January 2015 in three government hospitals in the Southern Nations, Nationalities, and People’s Region (SNNPR) of Ethiopia: Adari Hospital, Hawassa University Referral Hospital, and Yirgalem Hospital. The former two hospitals are situated in Hawassa (the regional capital city) and the latter is located 70 km from the city. Hawassa University Referral Hospital and Yirgalem Hospital are among the largest hospitals in southern Ethiopia and provide medical education and training in addition to medical care. The nature and type of medical waste released from these hospitals vary depending on their capacities and type of services they provide.

### Study population

The study population consisted of all MWH who worked in the three hospitals during the data collection period, comprising a total of 152 MWH. In order to control the effect of waste handling on exposure to hepatitis virus infection, 82 NMWH randomly selected from the main campus of Hawassa University were also included in this study. The NMWH were workers who clean classrooms, administrative offices, dining rooms, and student bedrooms and handle non-medical waste generated from these areas such as paper, dust particles, leftover foods, dead plant tissues and other non-clinical waste. MWH, who were on leave during the study period, did not consent to participation, or who had had contact with blood or other body fluids for less than six months within the previous year were excluded.

### Data collection

After obtaining informed written consent from each participant, data on sociodemographic characteristics and occupational and non-occupational risk factors for hepatitis virus exposure were collected using structured questionnaires.

### Laboratory testing

About 5 mL of venous blood was collected from all study participants according to the standard blood collection procedure. Separated sera were transported to the laboratory of Hawassa University Referral Hospital using a cold box and stored at -20°C until tested. All serum samples from MWH were screened for HBsAg, hepatitis B core antibody (total anti-HBc), and hepatitis B surface antibody (anti-HBs) using rapid diagnostic kits (Insight HBsAg; HBcAb phase IIC; HBsAb phase IIC; Tulip Diagnostics Pvt. Ltd., Goa, India). NMWH sera were screened only for HBsAg and anti-HBc markers. All tests were performed according to the manufacturer’s instructions and samples positive for either HBV or HCV sero-markers were rechecked by the same method.

### Definitions

Current HBV infection: MWH whose blood is serologically positive for HBsAg.

Life time exposure rate to HBV infection: MWH whose blood is serologically positive for anti-HBc following a natural infection and normally persists for life. Its presence may indicate a current or past resolved infection.

### Data analysis

Data entry and analysis was performed using SPSS version 16.0 (SPSS Inc., Chicago, IL, USA). Results were summarized using descriptive statistics. Multivariate logistic regression analysis was performed, by taking those sociodemographic and risk factors found to be significantly associated with lifetime exposure in bivariate logistic regression analysis. Odds ratios (OR) with 95% confidence intervals (CI) were calculated to measure the strength of associations, and *p*-values less than 0.05 were considered statistically significant.

### Ethical considerations

The study was approved by the institutional review board (IRB) of the Hawassa University College of Medicine and Health Sciences. A support letter was also obtained from the SNNPR Health Bureau. Participation in the study was fully voluntary and informed written consent was confirmed by the IRB. Participants were assured that information obtained in the course of the study would be kept confidential. All laboratory testing was performed free of charge and individuals positive for HBsAg or anti-HCV were managed by physicians. Furthermore, on-site training was given to MWH on how to handle, transport, and dispose of medical waste.

## RESULTS

### Sociodemographic characteristics

Out of the 200 MWH in the three government hospitals, 156 were eligible and approached for enrollment. Of these, 152 (97.4%) consented to participate in the study. The mean age of the MWH was 31.6 years (standard deviation [SD], 8.3; range, 18 to 60 years) while the mean age of NMWH was 28 years (SD, 8.1; range, 18 to 56 years). The female to male ratio was 8.5:1 in MWHs, while all NMWH were female. The majority of the MWH was married (67.1%) and had attended school to grade 7 to 10 (41.4%). The MWH had served in their current job for a mean of 5.5 years (range, 6 months to 30 years) and the majority (67.1%) were working at Hawassa University Referral Hospital (Table 1).

### Seroprevalence of hepatitis B virus and hepatitis C virus infection

As presented in Table 2, the prevalence of HBsAg, anti-HBc, and anti-HCV in MWH was 1.3%, 39.4%, and 0.7%, respectively. MWH had a significantly higher rate of lifetime exposure to HBV infection compared to NMWH (OR, 3.17; 95%CI, 1.64 to 6.13, p=0.001).

### Hepatitis B virus exposure rate and immunization status

The interpretation of results for various markers for HBV is presented in Table 3. The majority of the MWH (58.6%) were susceptible to HBV infection, while 22.4% developed immunity as a result of natural infection. Eleven MWH (7.2%) reported receiving at least one dose of hepatitis B vaccine; three of these individuals (27.3%) showed serological evidence of immunity (positive for anti-HBs).

**Figure 1.**
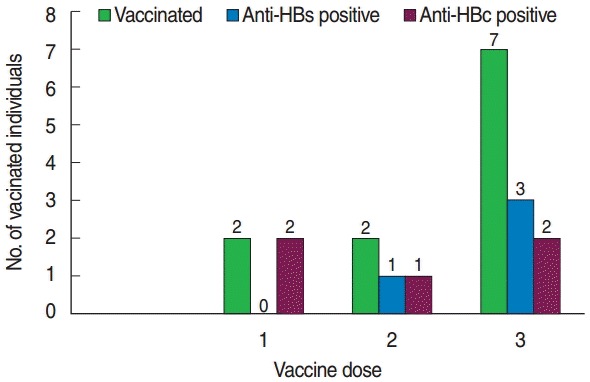
Frequency of HBV vaccinated MWHs with anti-HBs and anti-HBc against vaccine dose in southern Ethiopia, 2015. HBV, hepatitis B virus; MWHs, medical waste handlers; anti-HBs, hepatitis B surface antibody; anti-HBs, hepatitis B core antibody

All individuals had received vaccination without being screened for anti-HBc or anti-HBs status. Of the seven individuals who received the full HBV vaccination course (three doses), only three individuals developed protective antibodies (anti-HBs). Anti-HBs was not detected in those who received only a single vaccination dose (Figure 1).

### Hepatitis B virus exposure in relation to sociodemographic characteristics

In bivariate logistic regression analysis the rate of lifetime exposure to HBV infection was significantly higher among MWH who were older than 40 years of age (OR, 2.63; 95% CI, 1.11 to 6.20) and working in a hospital laundry (OR, 9.41; 95% CI, 1.06 to 83.84). However, other factors such as sex, marital status, educational level, years of service, and working hospital were not shown to influence HBV exposure status in MWH (Table 4). Moreover, none of the sociodemographic characteristics of NMWH were significantly associated with HBV exposure status.

### Factors associated with hepatitis B virus exposure

The association of HBV exposure with various occupational and non-occupational factors is described in Table 5. The rate of lifetime exposure to HBV infection was significantly higher among MWH who had received a tattoo (OR, 2.40; 95% CI, 1.07 to 5.41) or had multiple sexual partners (OR, 2.62; 95% CI, 1.00 to 6.87). No significant association was observed between HBV exposure and factors such as a history of work-related sharp injuries, mucosal exposure, surgical operations, liver disease, tooth extraction, blood transfusion, or a family history of liver disease (Table 5). Likewise, among NMWH, non-occupational factors such as surgical operations, liver disease, tooth extraction, blood transfusion, and family history of liver disease had no significant association with HBV exposure status.

In this study, 72 (47.4%) MWH had had at least one accidental sharp injury. Of these, 24 (33.3%) showed serological evidence of lifetime exposure to HBV infection. The identified causes of accidental sharp injury were improperly discarded needle and sharps (n=60, 83.3%), overfilled sharps containers (n=7, 9.7%), and improper handling of sharps during transportation (n=21, 29.1%). Only 48 (66.7%) of those with sharp injuries had reported their exposure to the responsible health professionals. Ten (20.8%) of these were tested for HIV infection, but none were tested for HBV or HCV infection. The failure to report accidents in 24 (33.3%) MWH was due to unawareness of the reporting procedure (n=6, 25%), lack of knowledge regarding available interventions (n=15, 62.5%), and a fear of learning or disclosing their serological status (n=3, 12.5%). Among the MWH, 17 (11.2%) individuals had experienced a splash of blood and body fluid to the eye, nose, or mucus membranes within the last year. Of these, 8 (47.1%) individuals showed serological evidence of lifetime exposure to HBV infection.

In multivariate logistic regression analysis, HBV exposure was increased in MWH older than 40 years of age compared with those less than 25 years of age (adjusted OR [aOR], 3.02; 95% CI, 1.09 to 8.39, p=0.034]. Moreover, MWH working in a hospital laundry had raised odds of lifetime HBV exposure compared to others working in the hospital wards (aOR, 10.34; 95% CI, 1.11 to 96.14, p=0.040).

### Compliance with universal precautions

All MWH were aware that medical waste contains harmful germs. However, only 58.6% of the study participants had ever heard of hepatitis virus transmission through medical waste, and only 7.2% were vaccinated. More than half (55.9%) of the MWH reported receiving no training on proper medical waste handling and infection prevention. Regarding the use of PPE, 96.1% of MWH reported donning heavy-duty gloves, 63.2% wore gowns, and 23% used a mask and apron consistently while handling medical waste. Moreover, only 9% of MWH used boots or closed-toed shoes, and none of MWH used goggles as protective equipment (data not shown).

## DISCUSSION

Occupational risk related to hepatitis virus exposure is a major concern for MWH in developing nations where a guideline for waste handling is not strictly followed. In this study, the prevalence of HBsAg, anti-HBc and anti-HCV in MWH was 1.3%, 39.4%, and 0.7%, respectively. MWH had a significantly higher rate of lifetime exposure to HBV infection compared to NMWH. The rate of HBV exposure was also shown to increase with age, such that participants older than 40 years had the highest rate of infection. In addition, MWH working in a hospital laundry were about ten-fold more likely to be exposed to HBV infection compared to others working in hospital wards. None of the other risk factors examined was found to influence the rate of HBV exposure.

The sero-prevalence reported in our study is in line with studies conducted among MWH in Tripoli, Libya that reported 2.3% prevalence of HBsAg (but also reported a higher rate of anti-HCV at 2.7%) [13] and in Palestine that reported 1.59% prevalence for HBsAg [14]. In contrast, higher rates have been reported in studies in Addis Ababa, central Ethiopia (6.3% for HBsAg) [11] and in Gondar, northwest Ethiopia (6% for HBsAg), although no difference was observed with respect to the rate of anti-HCV (1%) [12].

The significantly higher rate of lifetime exposure to HBV infection among MWH in comparison with NMWH in the current study this is in agreement with a study conducted in Addis Ababa [11] where MWH had 1.5 times the rate of exposure of NMWH. Moreover, MWH older than 40 years of age were also at increased odds of exposure to HBV infection in previous studies [11,13,15]. This increase with age may be due to the increased risk of exposure to HBV infection with time. The preponderance of HBV exposure among MWH based in hospital laundries may be due to the higher risk of contact with blood and other body fluids while handling contaminated materials.

Participants in our study who had tattoos or who had multiple sexual partners appeared to be at increased risk of lifetime exposure to HBV infection; however, this difference was not statistically significant after adjusting for other associated variables. Likewise, a trend towards a higher rate of HBV exposure among males compared with females was observed, but this was also not statistically significant. MWH with greater than 10 years of service appeared to be more affected by HBV compared to those with 1 years to 5 years of service, although the difference was not statistically significant. Previous studies of HCW indicate that the risk of exposure to HBV infection increases with longer service years [10,11,16,17].

Several other factors, such as prior blood transfusion, needle stick injuries, lack of training in infection prevention, and not being vaccinated, have been reported to increase the risk of HBV infection [15-19]. However, none of these associations was evident in our study. These days, most of the local blood bank centers screen blood for infections such as HIV, HBV, and HCV to prevent transfusion-transmitted infections. However, the frequent occurrence of sharp injuries among most MWH in the current and previous studies [16-20] highlights the need to give due attention to reducing such incidents. Causes of sharps injuries include improperly discarded sharps, improper handling of sharps during transportation, and handling over-filled sharps containers [21]. Improper disposal of sharps may be due to inadequate training, supervision, and support with regard to infection control and effective waste disposal practice provided to MWH. Resource shortages and workload pressures may also contribute to the failure to ensure the proper disposal of hazardous waste.

In this study, blood and other body fluids were involved in 11.2% of mucosal exposures within the last 12 months. This is significantly lower than the studies conducted in Addis Ababa (67.5%) [19], Gondar (64%) [22], Uganda (41%) [8] and Iran (86%) [23]. This disparity is perhaps due to differences in the sites of exposure: mouth, nose, eye and non-intact skin were considered in our study, while other studies also included intact skin on the hands and legs. Moreover, underreporting of exposures may result from employees’ fear of reprisal and job discrimination, or from recall bias.

Protective measures such as puncture-resistant sharps containers and extensive training and supervision for MWH on appropriate utilization of PPE such as gown, gloves, mask, apron, boots, and eye protection may reduce the incidence of sharps injuries and splash exposures. However, this key worker group will remain at risk until there is further improvement in the standards of waste segregation and disposal by healthcare professionals [21]. In this study, compliance with universal precautions was relatively high compared to other studies [12,17], but the frequent occurrence of sharps injuries and splash exposures indicates the inadequate utilization of PPE and highlights the need to strengthen training and supervision.

In this study, 39.4% of MWH were exposed to HBV infection; 22.4% of these developed protective immunity. However, the majority (58.9%) remained susceptible to infection and could potentially benefit from vaccination. Vaccination coverage was very low (7.2%) compared to countries like Turkey (27.5%) [24], Libya (21%) [13], and the UK (21%) [21]. This necessitates efforts from concerned bodies to enhance the availability of free vaccination against HBV infection for MWH.

This study was conducted in only three hospitals using a nonprobability sampling method at a single point in time. Therefore, selection and recall bias may have been introduced that might hinder the generalizability of the results to all MWH in the study area. Moreover, it was difficult for us to know whether the observed infections occurred before or after employment as MWH, since there was no medical screening at the time of employment. On the other hand, screening of NMWH to control the effect of occupational exposure to hepatitis virus infection may ensure quality in the generated data.

The prevalence of lifetime exposure to HBV infection among MWH was high, while the rate of HCV exposure was low. MWH older than 40 years and those working in hospital laundries were disproportionally infected. Almost two-thirds of MWH did not have protective immunity and were susceptible to HBV infection. Thus, screening upon hire, followed by vaccination of MWH is recommended to reduce the transmission of HBV. Moreover, the need for training in infection prevention is emphasized, particularly in relation to waste handling and disposal.

## Figures and Tables

**Table 1.** Sociodemographic characteristics of MWH and NMWH in southern Ethiopia, 2015

Characteristic		MWH (n = 152)	NMWH (n = 82)
Sex	Male	16 (10.5)	0 (0.0)
	Female	136 (89.5)	82 (100)
Age (yr)	18-28	67 (44.1)	51 (62.2)
	29-39	52 (34.2)	22 (28.8)
	≥40	33 (21.7)	9 (11.0)
Marital status	Married	102 (67.1)	41 (50.0)
	Single	13 (8.6)	28 (34.1)
	Divorced	24 (15.8)	6 (7.3)
	Widowed	13 (8.6)	7 (8.5)
Educational level	Illiterate	31 (20.4)	4 (4.9)
	1-6 grade	58 (38.2)	24 (29.3)
	7-10 grade	63 (41.4)	42 (51.2)
	Certificate	N/A	12 (14.7)
Years of service	<1	38 (25.0)	23 (28.0)
	1-5	36 (23.7)	59 (72.0)
	5-10	60 (44.7)	0 (0.0)
	>10	10 (6.6)	0 (0.0)
Working hospital	HURH	102 (67.1)	82 (100)^1^
	Yirgalem Hospital	28 (18.4)	N/A
	Adari Hospital	22 (14.5)	N/A

Values are presented as number (%). MWH, medical waste handlers; NMWH, non-medical waste handlers; N/A, not applicable; HURH, Hawassa University Referral Hospital.

1 Hawassa University main campus.

**Table 2.** Distribution of hepatitis B and C virus markers in MWH and NMWH in southern Ethiopia, 2015

Hepatitis virus markers	No. of positive individuals		OR (95%CI)	p-value
	MWH (n = 152)	NMWH (n = 82)		
HBsAg	2 (1.3)	2 (2.4)	0.53 (0.07, 3.86)	0.53
Anti-HBc	60 (39.4)	14 (17.1)	3.17 (1.64, 6.13)	0.001
Anti-HCV	1 (0.7)	1 (1.2)	0.54 (0.03, 8.69)	0.66

Values are presented as number (%). NMWH, non-medical waste handlers; MWH, medical waste handlers; OR, odds ratio; CI, confidence interval; HBsAg, hepatitis B surface antigen; Anti-HBc; hepatitis B core antibody; Anti-HCV, antibody hepatitis C virus.

**Table 3.** Interpretation of serologic markers: hepatitis B virus infection status and corresponding percentages among medical waste handlers in southern Ethiopia, 2015

Serologic marker			Interpretation	n (%)
HBsAg	Anti-HBc	Anti-HBs		
Negative	Negative	Negative	Susceptible	89 (58.6)
Negative	Positive	Positive	Immune after infection	34 (22.4)
Negative	Negative	Positive	Immune after vaccination	3 (2.0)
Positive	Positive	Negative	Current infection	2 (1.3)
Negative	Positive	Negative	Indeterminate: four possibilities	24 (15.7)
			- Resolving infection (window phase)	
			- Remote resolved infection with low anti-HBs	
			- Chronic infection with low levels of HBsAg	
			- False positive anti-HBc, hence susceptible.	

HBsAg, hepatitis B surface antigen; Anti-HBc, hepatitis B core antibody; Anti-HBs, hepatitis B surface antibody.

**Table 4.** Bivariate analysis of sociodemographic characteristics by lifetime exposure to hepatitis B virus infection in MWH in southern Ethiopia, 2015

Characteristic		MWH	Lifetime exposure	Unadjusted OR (95% CI)	p-value
Sex	Male	16 (10.5)	7 (43.8)	1.22 (0.43, 3.47)	0.71
	Female	136 (89.5)	53 (39.0)	1.00 (reference)	
Age (yr)	18-25	43 (28.3)	13 (30.2)	1.00 (reference)	
	26-39	76 (50.0)	29 (38.2)	1.42 (0.64, 3.16)	0.39
	≥40	33 (21.7)	18 (54.5)	2.77 (1.08, 7.12)	0.03
Marital status	Married	102 (67.1)	42 (41.2)	2.33 (0.60, 8.99)	0.22
	Single	13 (8.6)	3 (23.1)	1.00 (reference)	
	Divorced/widowed	37 (24.3)	15 (40.5)	2.27 (0.53, 9.66)	0.27
Educational level	Illiterate	31 (20.4)	13 (41.9)	1.18 (0.49, 2.87)	0.71
	1-6 grade	58 (38.2)	22 (37.9)	1.00 (reference)	
	7-10 grade	63 (41.4)	25 (39.7)	1.08 (0.52, 2.24)	0.83
Years of service	<1	24 (15.8)	12 (50.0)	1.47 (0.60, 3.60)	0.39
	2016-01-05	45 (29.6)	13 (28.9)	1.00 (reference)	
	2016-05-10	68 (44.7)	25 (36.8)	1.04 (0.46, 2.35)	0.93
	>10	15 (9.9)	10 (67.7)	3.57 (0.30, 42.99)	0.32
Working hospital	HURH	102 (67.1)	37 (36.3)	1.00 (reference)	
	ADH	22 (14.5)	9 (40.9)	1.22 (0.47, 3.11)	0.68
	YAH	28 (18.4)	14 (50.0)	1.76 (0.76, 4.08)	0.19
Working environment	Ward	98 (64.5)	34 (34.7)	1.00 (reference)	
	OPD	34 (22.4)	14 (41.2)	1.32 (0.59, 2.93)	0.50
	Laboratory	9 (5.9)	4 (44.4)	1.51 (0.38, 5.98)	0.56
	Laundry	6 (3.9)	5 (83.3)	9.41 (1.06, 83.84)	0.04
	ICU	5 (3.3)	3 (60.0)	2.82 (0.45, 17.72)	0.27

Values are presented as number (%). MWH, medical waste handlers; OR, odds ratio; CI, confidence interval; HURH, Hawassa University Referral Hospital; ADH, Adari Hospital; YAH, Yirgalem Hospital; OPD, outpatient department; ICU, intensive care unit.

**Table 5.** Bivariate analysis of occupational and non-occupational risk factors by lifetime exposure to hepatitis B virus infection in MWH in southern Ethiopia, 2015

Characteristic		MWH	Lifetime exposure	Unadjusted OR (95% CI)	p-value
Sharp injuries	Yes	72 (47.4)	24 (33.3)	1.00 (reference)	
	No	80 (52.6)	36 (45.0)	1.64 (0.85, 3.16)	0.14
Mucosal exposure	Yes	17 (11.2)	8 (47.1)	1.42 (0.51,3.91)	0.50
	No	135 (88.8)	52 (38.5)	1.00 (reference)	
Surgical operation	Yes	25 (16.4)	12 (48.0)	1.52 (0.64, 3.60)	0.34
	No	127 (83.6)	48 (37.8)	1.00 (reference)	
Blood transfusion	Yes	3 (2.0)	1 (33.3)	1.00 (reference)	
	No	148 (98.0)	59 (39.6)	1.31 (0.12, 14.79)	0.83
History of liver disease	Yes	3 (2.0)	2 (66.7)	3.14 (0.28, 35.39)	0.35
	No	149 (98.0)	58 (38.9)	1.00 (reference)	
Tattooing	Yes	30 (19.7)	17 (56.7)	2.40 (1.07, 5.41)	0.03
	No	122 (80.3)	43 (35.2)	1.00 (reference)	
Tooth extraction	Yes	46 (30.3)	22 (47.8)	1.64 (0.81,3.31)	0.17
	No	106 (69.7)	38 (35.8)	1.00 (reference)	
Multiple sexual partners	Yes	20 (13.2)	12 (60.0)	2.62 (1.00, 6.87)	0.05
	No	132 (86.8)	48 (36.4)	1.00 (reference)	
Family history of liver disease	Yes	5 (3.3)	2 (40.0)	1.02 (0.17, 6.31)	0.98
	No	147 (96.7)	58 (39.5)	1.00 (reference)	

Values are presented as number (%). MWH, medical waste handlers; OR, odds ratio; CI, confidence interval.
